# The Fiber Cell-Specific Overexpression of *COMT2* Modulates Secondary Cell Wall Biosynthesis in Poplar

**DOI:** 10.3390/plants14121739

**Published:** 2025-06-06

**Authors:** Hanyu Chen, Hong Wang, Zhengjie Zhao, Jiarui Pan, Yao Yao, Yihan Wang, Keming Luo, Qin Song

**Affiliations:** 1 Key Laboratory of Eco-Environments of Three Gorges Reservoir Region, Ministry of Education, Chongqing Key Laboratory of Tree Germplasm Resource Innovation and Utilization, School of Life Sciences, Southwest University, Integrative Science Center of Germplasm Creation in Western China (Chongqing) Science City, Chongqing 400715, China; gutou289@163.com (H.C.); zzj112022705385278@email.swu.edu.cn (Z.Z.); pjrokk@126.com (J.P.); yaoyao041016@126.com (Y.Y.); 18843349120@163.com (Y.W.); 2 College of Agronomy and Biotechnology, Southwest University, Chongqing 400715, China; wh059260@163.com

**Keywords:** poplar, *COMT2*, SCW, lignin, mechanical strength

## Abstract

Wood, as a natural and renewable resource, plays a crucial role in industrial production and daily life. Lignin, as one of the three major components of the plant cell secondary wall, plays a key role in conferring mechanical strength and enhancing stress resistance. The caffeic acid-O-methyltransferase (COMT) family of oxygen-methyltransferases is a core regulatory node in the downstream pathway of lignin biosynthesis. Here, our report shows that *caffeic acid-O-methyltransferase 2* (*COMT2*) exhibits high conservation across several species. Tissue expression analysis reveals that *COMT2* is specifically highly expressed in the secondary xylem of *Populus tomentosa* stems. We demonstrated that the specific overexpression of *COMT2* in fiber cells of *Populus tomentosa* led to a significant increase in plant height, stem diameter, internode number, and stem dry weight. Furthermore, we found that the specific overexpression of *COMT2* in fiber cells promotes xylem differentiation, lignin accumulation, and the thickening of the secondary cell wall (SCW) in fiber cells. Our results indicate that key downstream lignin biosynthesis enzyme genes are upregulated in transgenic plants. Additionally, mechanical properties of stem bending resistance, puncture resistance, and compressive strength in the transgenic lines are significantly improved. Moreover, we further created the *DUF*pro:*COMT2* transgenic lines of *Populus deltoides* × *Populus. euramericana* cv ‘Nanlin895’ to verify the functional conservation of *COMT2* in closely related poplar species. The *DUF*pro:*COMT2 Populus deltoides* × *Populus. euramericana* cv ‘Nanlin895’ transgenic lines exhibited phenotypes similar to those observed in the *P. tomentosa* transgenic plants, which showed enhanced growth, increased lignin accumulation, and greater wood strength. Overall, the specific overexpression of the caffeic acid O-methyltransferase gene *COMT2* in poplar stem fiber cells has enhanced the wood biomass, wood properties, and mechanical strength of poplar stems.

## 1. Introduction

The cell wall, analogous to an “exoskeleton” in plant cells, plays a critical role in the process of cell morphogenesis, determining the morphogenesis of plant organs and even the entire plant [1]. The plant cell wall is of profound significance, serving not only as the primary source of dietary fiber but also providing fundamental raw materials for traditional industries such as textile production, wood processing, and pulping and papermaking. Additionally, it exhibits substantial potential application value in the field of biofuels [2]. The secondary cell wall (SCW), primarily present in the tracheary elements of vascular plants, including vessels, tracheids, and xylem fibers [3], provides mechanical support to plants, enabling vascular plants to grow vertically. Additionally, the SCW allows the conductive tissues to resist the negative pressure generated by transpiration while preventing water loss within these tissues, thus facilitating efficient water transport [3]. The SCW, formed subsequent to the cessation of plant cell expansion, exhibits a greater thickness and higher mechanical strength when compared to the primary cell wall [4]. In woody plants, wood biomass predominantly consists of SCWs, which constitute the principal structural components of fibers and tracheary elements in plants. The biosynthesis of these cell wall components is meticulously coordinated during the process of secondary wall formation. A majority of the genes implicated in the lignin biosynthetic pathway have been successfully isolated and functionally characterized in the poplar plants [5]. The constituent components of the SCW primarily consist of lignin, cellulose, and hemicellulose [6]. Although the composition and basic functions of the SCW are well studied, the complexity of its biosynthetic regulatory network remains poorly understood. Recent research shows that its biosynthesis is precisely regulated by transcription factors, such as NAC and MYB family members. For example, NAC proteins staphylococcal nuclease and tudor domain-containing 1 (SND1) and vascular-related NAC-domain6 (VND6) activate genes encoding cellulose synthases (CesA) and lignin biosynthetic enzymes, such as phenylalanine ammonia-lyase(PAL) and 4-coumarate:CoA ligase (4CL), and coordinate spatiotemporal deposition of wall components [7,8]. Several MYB transcription factors form regulatory cascades with NACs to fine-tune secondary wall thickness and chemical composition [9]. These findings reveal multi-layered regulatory mechanisms of SCW formation, though their connection to lignin structural diversity remains unexplored.

Lignin, one of the central end products of the plant phenylpropanoid metabolic pathway, initiates its biosynthesis through the deamination of phenylalanine to generate the key precursor p-coumaric acid [10]. As a major structural component of the SCWs in vascular plants, lignin constitutes the second most abundant biopolymer in biomass after cellulose, accounting for approximately 30% of the organic carbon storage in the terrestrial biosphere [11]. The biosynthesis of lignin and its metabolic regulatory network are of critical importance for plant growth and development. As a complex phenolic polymer, lignin maintains the structural integrity of vascular bundles by enhancing cell wall rigidity and hydrophobicity, thereby facilitating the directional transport of minerals [12]. Simultaneously, lignin forms a physicochemical barrier against pest and pathogen infection by reinforcing the cell wall and releasing resistant compounds [13,14]. Additionally, lignin biosynthesis directly enhances plant lodging resistance by reinforcing cell wall mechanical strength while participating in the adaptive responses to abiotic stresses such as drought and salinity through regulating secondary wall components [15,16,17]. The functions collectively highlight the central role of lignin in plant growth and environmental adaptation.

O-methyltransferases (OMTs), as a major group of enzymes, are responsible for catalyzing the methylation modification of oxygen atoms in phenylpropanoids, flavonoids, and certain alkaloids [18,19]. Their catalytic products not only participate in lignin biosynthesis but also play important roles in the generation of pharmacologically active substances, antimicrobial defense (phytoalexins), stress tolerance, and ecological interactions [20]. Plant OMTs can be divided into two major evolutionary clades: Clade I includes caffeoyl-CoA O-methyltransferase (CCoAOMT) and COMT, while Clade II encompasses all other OMT types. In the lignin biosynthesis pathway of dicotyledonous plants, the two enzymes in Clade I perform specific catalytic functions: CCoAOMT participates in the synthesis of guaiacyl (G-type) lignin by methylating the p-hydroxy group of hydroxycinnamoyl-CoA esters, whereas COMT catalyzes the aliphatic hydroxyl methylation of caffeic acid and 5-hydroxyferuloyl-CoA, directing the biosynthesis of syringyl (S-type) lignin. Notably, silencing the *Populus* COMT gene results in a significant reduction in total lignin content, accompanied by substantial changes in lignin structure: the content of condensed bonds increases twofold, S-type lignin is nearly completely depleted, while G-type lignin levels rise significantly [21]. These findings indicate that COMT exerts precise regulatory effects on the chemical structure of lignin. In *Taxus* trees, COMT family genes are primarily highly expressed in the xylem. Among them, *CbuCOMT23* is significantly downregulated in tension wood but upregulated in opposite wood, suggesting their responsiveness to mechanical stress and involvement in the adaptive regulation of wood mechanical properties [22]. Studies have shown that *COMT2* exhibits the highest expression level in the differentiating xylem of *Populus trichocarpa* [23], suggesting a potential role in secondary wall synthesis. However, current reports are limited regarding the specific regulation of *COMT2* expression in stem fiber cells and its effects on lignin biosynthesis and stem strength in poplar stems.

In this study, we generated transgenic *P. tomentosa* plants in which *COMT2* was driven by the fiber cell-specific promoter *DUF759*. Our results showed that fiber cell-specific overexpression of *COMT2* promoted secondary xylem differentiation, lignin biosynthesis, and the bending, puncture, and extrusion resistance of poplar wood. Furthermore, this function was conserved in *Populus deltoides × Populus euramericana ‘Nanlin895’*. This study validated a novel role for *COMT2* in wood development and SCW biosynthesis, providing new insights for tree breeding.

## 2. Results

### 2.1. Characterization of COMT2 from Populus Tomentosa

**Figure 1:**
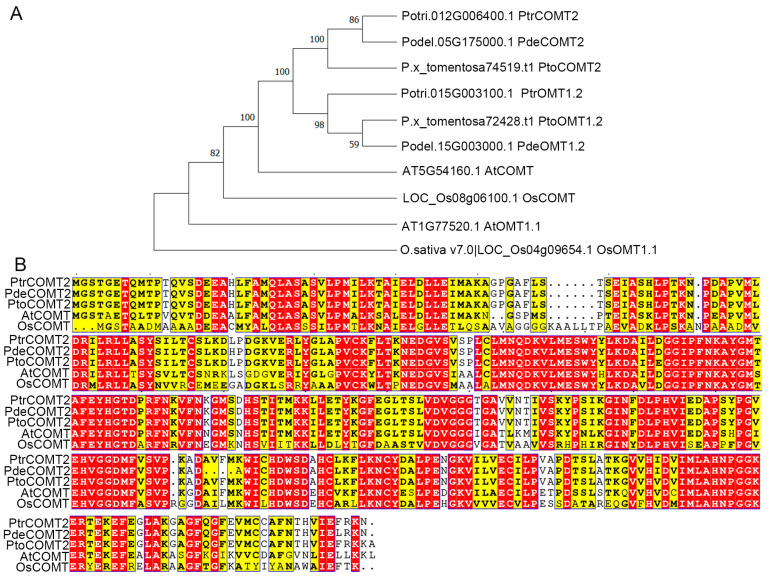
Phylogenetic analysis and amino acid alignment of *COMT2*. (**A**) Phylogenetic relationship of *COMT2* from *A. thaliana*, *O. sativa*, *P. deltoides*, *P. trichocarpa* and *P. tomentosa*. (**B**) Amino acid alignment of *COMT2*. A red background indicates highly conserved residues, while a yellow background indicates similar residues.

The 1095 bp full-length ORF sequence of *COMT2* encoding a 39.82 kDa protein was cloned from the cDNA of *P. tomentosa*. The COMT2 protein shares an overall identity of 90.41% with the homolog protein (P.x_tomentosa72428.t1). To investigate whether there are differences in the evolutionary relationships of the *COMT2* amino acid sequences among *Arabidopsis thaliana*, *Oryza sativa*, *Populus trichocarpa*, *Populus deltoides*, and *Populus tomentosa*, this study constructed a phylogenetic tree based on the *COMT2* amino acid sequences of these species. The results revealed that the evolutionary distances of *COMT2* among different species are relatively close (Figure 1A). Multiple sequence alignment of COMT amino acid sequences demonstrated a consensus level of 71.51%, with the similarity of *COMT2* amino acid sequences reaching as high as 90.16% (Figure 1B). These findings suggest that *COMT2* is likely highly conserved across *Arabidopsis thaliana*, *Oryza sativa*, *Populus trichocarpa*, *Populus deltoides*, and *Populus tomentosa*.

**Figure 2:**
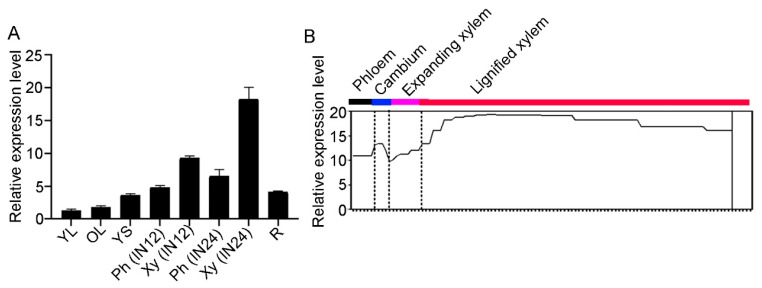
The expression pattern of *COMT2* in *P. tomentosa.* (**A**) Relative expression levels of *COMT2* were determined by RT-qPCR in different tissues of *P. tomentosa*. YL means young leaf; OL means old leaf; YS means young stem; Ph(IN12) means phloem of the 12th internode; Xy(IN12) means xylem of the 12th internode; Ph(IN24) means phloem of the 24th internode; Xy(IN24) means xylem of the 24th internode; R means root. (**B**) The expression pattern analysis of *COMT2* in stems of poplar. Data from Aspwood (http://aspwood.popgenie.org), accessed on 13 December 2019. The dotted line indicates the separation of different tissue positions in the stem.

To investigate the expression pattern of *COMT2* in various tissues of *P. tomentosa*, we collected young leaves, mature leaves, roots, young stems (internodes 1 to 3), xylem and phloem of the 12th internode (IN12), and xylem and phloem of the 24th internode (IN24) from two-month-old greenhouse-cultivated WT *P. tomentosa* plants. Total RNA was extracted from each tissue and reverse-transcribed into cDNA. RT-qPCR analysis revealed that *COMT2* exhibits higher expression levels in developing and mature xylem, with its expression increasing progressively during secondary development (Figure 2A). Using the ASPWOOD database (http://aspwood.popgenie.org/aspwood-v3.0/), accessed on 13 December 2019, the expression patterns of *COMT2* in different tissues of the stem were analyzed, revealing its high expression in the xylem (Figure 2B). These results suggest that *COMT2* may play an important role in wood development.

### 2.2. Generation of Fiber Cells Specific Overexpression of COMT2 Transgenic Lines in P. tomentosa

**Figure 3:**
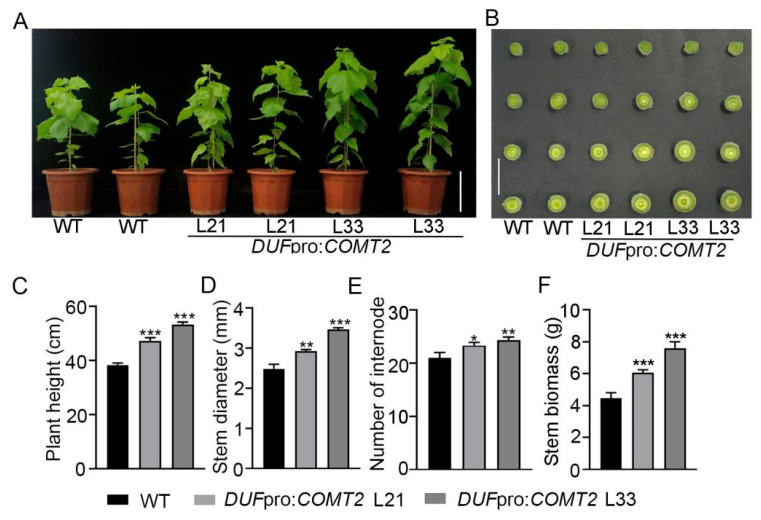
Phenotype analysis of *DUF*pro:*COMT2* transgenic plants in *P. tomentosa.* (**A**) Phenotypes of two-month-old WT, *DUF*pro:*COMT2* poplar plants. Bar = 15 cm. (**B**) Comparison of cross-sections of different parts of stems between WT and transgenic poplar trees. Bar = 1 cm. (**C**–**F**). Plant height (**C**), stem diameter (**D**), number of internode (**E**) and stem biomass (**F**) of two-month-old WT, *DUF*pro:*COMT2 P. tomentosa* transgenic plants. Student’s *t*-test: * *p* < 0.05; ** *p* < 0.01; *** *p* < 0.001; *n* = 3.

To validate the function of *COMT2* in wood fiber cells, *COMT2* was overexpressed in *P. tomentosa* under the control of the fiber-specific promoter *DUF579-9*. Eight independent overexpression lines (*DUF*pro:*COMT2*) with high expression levels of *COMT2* were identified via PCR amplification (Figure S1A) and qRT-PCR (Figure S1B). Two lines with the highest expression levels, L21 and L33, were selected for further investigation. Phenotypic observations of two-month-old WT and *DUF*pro:*COMT2* transgenic *P. tomentosa* plants revealed that the transgenic plants exhibited superior growth (Figure 3A). Additionally, comparative analysis of stem cross-sections at four marked positions indicated that the stem diameter of transgenic lines was increased compared to WT (Figure 3B). Overexpression of *COMT2* in transgenic plants resulted in significant increases in plant height (Figure 3C), stem diameter (Figure 3D), and internode number (Figure 3E). Specifically, compared to WT plants, the stem dry weight of L21 and L33 lines increased by 39.07% and 60.58%, respectively (Figure 3F). These findings demonstrate that the wood fiber-specific overexpression of the *COMT2* gene promotes overall growth in *P. tomentosa*.

### 2.3. COMT2 Positively Regulates Xylem Development During Wood Formation in P. tomentosa

**Figure 4:**
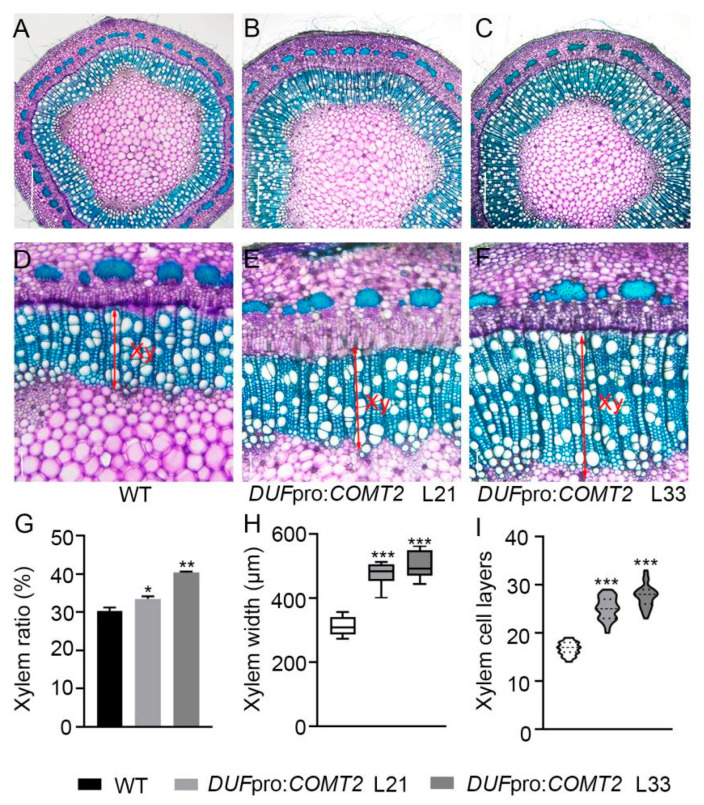
*COMT2*-dependent regulation on xylem cell development in *P. tomentosa.* (**A**–**F**) Cross-sections of the marked stem internode were stained with toluidine blue from 2-month-old WT and *DUF*pro:*COMT2 P. tomentosa* transgenic plants. Bar (**A**–**C**) = 500 μm; bar (**D**–**F**) = 100 μm. (**G**–**I**) Measurement and quantification of percentage of secondary xylem in the whole stem (**G**), xylem width (**H**) and secondary xylem cell layers (**I**). Student’s *t*-test: *, *p* < 0.05; **, *p* < 0.01; ***, *p* < 0.001; *n* = 3. Xy means xylem.

To identify its role of *COMT2* in regulating secondary xylem development during wood formation. Cross-sections were generated from the second marked internode in two-month-old WT and *DUF*pro:*COMT2* transgenic *P. tomentosa* plants and stained by toluidine blue O (Figure 4A–F). Secondary xylem development was significantly increased by overexpressing *COMT2* in fiber cells of *P. tomentosa* (Figure 4A–F), as evidenced by increased xylem occupancy (Figure 4G) and xylem width (Figure 4H) in the stem. Compared with WT, the number of xylem cell layers increased 50–65% in transgenic poplars (Figure 4I). These results indicate that *COMT2* promotes xylem differentiation in poplar plants.

### 2.4. Fiber Cells Specific Overexpression of COMT2 Promotes SCW Deposition and Stem Strength in P. tomentosa

**Figure 5:**
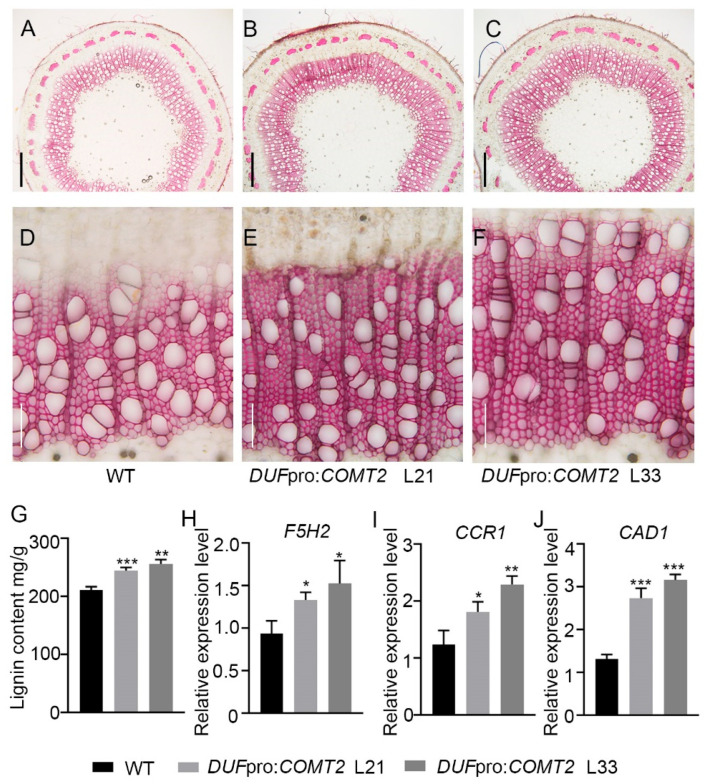
*COMT2*-dependent regulation on lignin deposition during wood formation in *P. tomentosa*. (**A**–**F**) Cross-sections of the marked stem internode were stained with phloroglucinol-HCl from 2-month-old WT and *DUF*pro:*COMT2 P. tomentosa* transgenic plants. Bar (**A**–**C**) = 500 μm; bar (**D**–**F**) = 100 μm. (**G**) Measurement and quantification of lignin content in the stem of WT and *DUF*pro:*COMT2 P. tomentosa* transgenic plants. (**H**,**I**) Expression analysis of lignin biosynthetic genes in WT and *DUF*pro:*COMT2 P. tomentosa* transgenic plants, including *F5H2* (**H**), *CCR1* (**I**) and *CAD1* (**J**). Student’s *t*-test: * *p* < 0.05; ** *p* < 0.01; *** *p* < 0.001; n = 3.

Phloroglucinol staining of stem cross-sections was further employed to investigate lignin deposition in WT and *DUF*pro:*COMT2* transgenic lines. (Figure 5A–F). The observations revealed that the transgenic lines exhibited a more intense red coloration compared to the WT (Figure 5A–F). To quantify the secondary xylem modifications, we measured the lignin content in the stem of WT and transgenic plants. Consistent with these observations, the lignin content of *DUF*pro:*COMT2* transgenic lines L21 and L33 was increased by 16% and 22%, respectively, compared with that of the WT (Figure 5G). Quantitative analysis of key enzyme genes involved in the downstream lignin biosynthesis pathway revealed that the expression levels of *Ferulate 5-hydroxylase 2* (*F5H2*) (Figure 5H), *cinnamoyl-CoAreductase 1* (*CCR1*) (Figure 5I), and *cinnamyl-alcohol dehydrogenase 1* (*CAD1*) (Figure 5J) were significantly upregulated in the overexpression lines. In conclusion, these findings indicate that *COMT2* promotes lignin biosynthesis in poplar plants.

**Figure 6:**
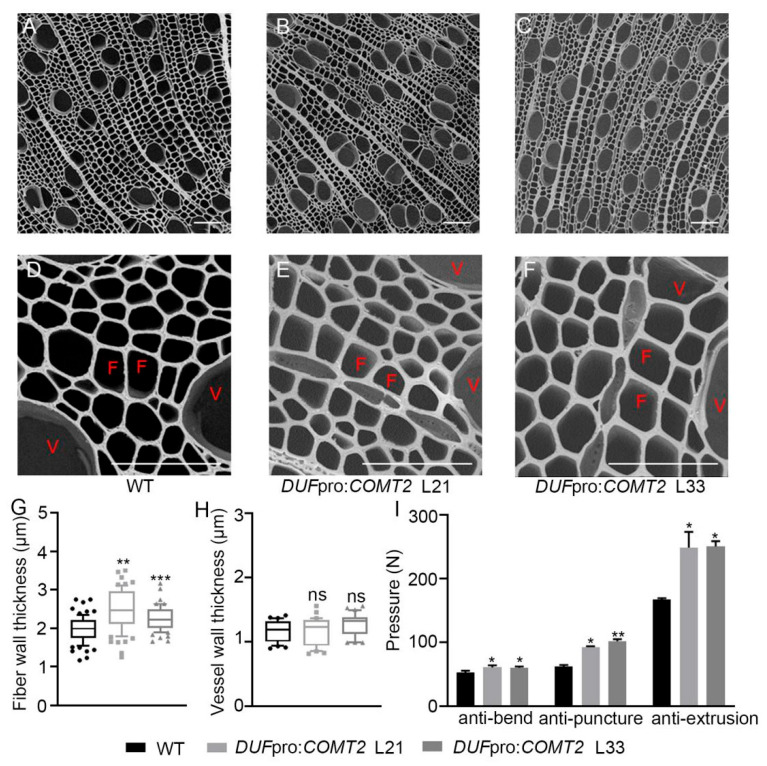
*COMT2*-dependent regulation of secondary wall deposition during wood formation in *P. tomentosa.* (**A**–**F**) A secondary wall of the marked stem internode was observed with scanning electron microscopy in WT and *DUF*pro:*COMT2 P. tomentosa* transgenic plants. Bar (**A**–**C**) = 100 μm; bar (**D**–**F**) = 20 μm. (**G**,**H**) The secondary cell wall thickness measurement of fiber cells (**G**) and vessel cells (**H**). (**I**) The pressure of anti-bend, anti-puncture and anti-extrusion in WT and *DUF*pro:*COMT2 P. tomentosa* transgenic plants. Student’s *t*-test: * *p* < 0.05; ** *p* < 0.01; *** *p* < 0.001; ns means no significant difference; n = 3. V means vessel cells; F means fiber cells.

Furthermore, tissue cross-sections of the 2nd marked internode were prepared and subjected to gradient dehydration for scanning electron microscopy (SEM) observation (Figure 6A–F). Statistical analysis revealed that the fiber cell walls of the L21 and L33 lines of *DUF*pro:*COMT2* were thickened by 21.25% and 26.93%, respectively (Figure 6G), while no significant difference was observed in the thickness of vessel cell walls (Figure 6H). As SCW biosynthesis directly enhances the lodging resistance of plants by increasing the mechanical strength of the cell walls [15]. Mechanical tests, including bending, puncture, and extrusion resistance, were conducted on the stems of WT and *DUF*pro:*COMT2* transgenic *P. tomentosa* lines. The results demonstrated that overexpression of *COMT2* enhanced the stem strength of poplar plants (Figure 6I). In conclusion, these findings indicate that the wood fiber-specific expression of *COMT2* significantly promotes lignin biosynthesis and secondary wall deposition in fiber cells of the secondary xylem and enhances the mechanical strength of poplar stems.

### 2.5. Generation of Fiber Cells Specific Overexpression of COMT2 Transgenic Lines in P. deltoides × P. euramericana cv ‘Nanlin895’

**Figure 7:**
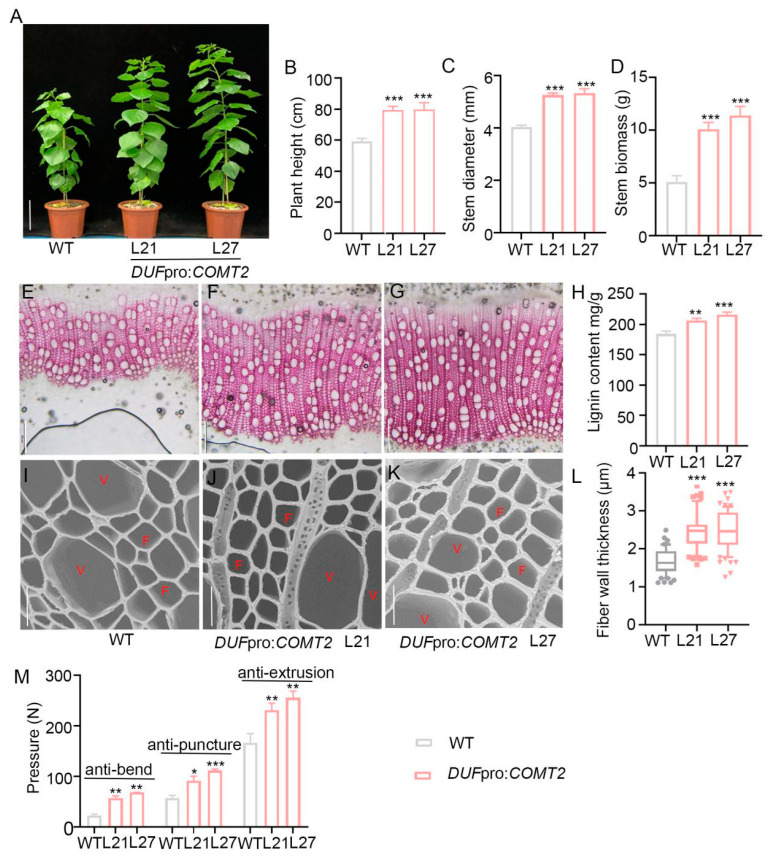
Morphological phenotypes of the fiber cells specific to overexpression of *COMT2* in *P. deltoides* × *P. euramericana* cv ‘Nanlin895’ poplar lines. (**A**) Phenotypes of two-month-old WT, *DUF*pro:*COMT2 P. deltoides* × *P. euramericana* cv ‘Nanlin895’ transgenic plants. Bar = 15 cm. (**B**–**D**). Plant height (**B**), stem diameter (**C**) and stem biomass (**D**) of two-month-old WT, *DUF*pro:*COMT2 P. deltoides* × *P. euramericana* cv ‘Nanlin895’ transgenic plants. (**E**–**G**) Cross-sections of the marked stem internode were stained with phloroglucinol-HCl from 2-month-old WT and *DUF*pro:*COMT2 P. deltoides* × *P. euramericana* cv ‘Nanlin895’ transgenic plants. Bar = 200 μm. (**H**) Measurement and quantification of lignin content in the stem of WT and *DUF*pro:*COMT2 P. deltoides* × *P. euramericana* cv ‘Nanlin895’ transgenic plants. (**I**–**K**) A secondary wall of the marked stem internode was observed with scanning electron microscopy in WT and *DUF*pro:*COMT2 P. deltoides* × *P. euramericana* cv ‘Nanlin895’ transgenic plants. Bar = 20 μm. (**L**) The secondary cell wall thickness measurement of fiber cells. (**M**) The pressure of anti-bend, anti-puncture and anti-extrusion in WT and *DUF*pro:*COMT2 P. deltoides* × *P. euramericana* cv ‘Nanlin895’ transgenic plants. Student’s *t*-test: * *p* < 0.05; ** *p* < 0.01; *** *p* < 0.001; n = 3. V means vessel cells; F means fiber cells.

To verify the conservation of *COMT2* in regulating SCW synthesis across different *Populus* species, the recombinant vector *DUF*pro:*COMT2* was introduced into *P. deltoides* × *P. euramericana* cv ‘Nanlin895’ plants using the *Agrobacterium*-mediated leaf disc transformation method. Following RT-qPCR identification, positive transgenic lines were obtained, and the L21 and L27 lines with higher expression levels were selected for subsequent experiments (Figure S2). Phenotypic observations of two-month-old WT and *DUF*pro:*COMT2* transgenic *P. deltoides* × *P. euramericana* cv ‘Nanlin895’ plants revealed that the transgenic plants also exhibited superior growth (Figure 7A). Measurement of plant height (Figure 7B), stem diameter (Figure 7C), and the increased stem dry weight (Figure 7D). Toluidine blue O staining (Figure S3A) and statistical analysis of xylem layer numbers (Figure S3B) in the 2nd marked internode revealed that *COMT2* also promotes xylem differentiation in *P. deltoides* × *P. euramericana* cv ‘Nanlin895’ poplars.

Similarly, we analyzed changes in lignin synthesis and secondary wall deposition in the stems of *DUF*pro:*COMT2* transgenic *P. deltoides* × *P. euramericana* cv ‘Nanlin895’ plants through phloroglucinol-HCl chemical staining (Figure 7E–G) and SEM morphological observations (Figure 7I–K). Lignin content measurement (Figure 7H) and statistical analysis (Figure 7L) of secondary wall thickness in fiber cells demonstrated that increased expression of *COMT2* in fiber cells promoted secondary wall deposition in the xylem of *P. deltoides* × *P. euramericana* cv ‘Nanlin895’ poplar stems. RT-qPCR analysis showed the expression levels of *F5H2* (Figure S4A), *CCR1* (Figure S4B), and *CAD1* (Figure S4C) were significantly upregulated in the overexpression lines. Additionally, the stem strength of transgenic poplar was significantly enhanced compared to that of the WT (Figure 7M), which was consistent with the results observed in *COMT2*-overexpressing lines of *P. tomentosa*.

Overall, our results demonstrate that the specific overexpression of *COMT2* in fiber cells promotes the expression of downstream lignin-related genes, thereby enhancing lignin synthesis and SCW deposition, ultimately leading to an increase in stem strength in poplar.

## 3. Discussion

Fast-growing poplar wood, a renewable resource, has significant economic value. As a major commercial tree species, it still has substantial potential for improving properties like yield, hardness, and mechanical strength. Lignin biosynthesis is central to secondary xylem development in poplar, providing wood with mechanical strength and wear resistance. Lignin content and structure directly affect key performance indicators, thus being pivotal for optimizing poplar wood quality. This study overexpressed *caffeoyl-CoA O-methyltransferase 2* (*COMT2*) in poplar stem fiber cells, enhancing lignin biosynthesis and secondary wall deposition in secondary xylem fiber cells of specific tissues. This approach generated new poplar germplasms with significantly improved plant growth, timber yield, and stem strength.

### 3.1. Genetic Functional Conservation of the COMT Family and Its Effects on Plant Growth

The *COMT* gene encodes caffeoyl-CoA O-methyltransferase (an O-methyltransferase), which plays a critical role in plant growth, development, and stress responses [24]. Previous studies have shown that among popular COMT family members, *COMT2* exhibits the highest transcriptional activity during xylem differentiation stages [23]. Consistent with previous reports, this study found that the *P. tomentosa COMT2* gene is highly expressed in poplar stems, with its expression showing a gradual increase as the internode number increases. This suggests that *COMT2* may play important regulatory roles in the secondary development of poplar. Phylogenetic analysis and amino acid sequence alignment of *COMT2* genes identified in *O. sativa*, *A. thaliana*, *P. trichocarpa*, *P. tomentosa*, and *P. deltoides* revealed high conservation of *COMT2* across these species. The *COMT2* gene mediates lignin biosynthesis methylation, regulating monolignol composition to influence cell wall strength and stress resistance. In tomato and poplar plants, *COMT2* was demonstrated to promote lignin accumulation and regulate fiber development under stress tolerance [25,26], indicating its preservation as a key adaptive gene in plant evolution. Our study utilized the xylem fiber cell-specific promoter *DUF579-9* to drive spatiotemporal-specific overexpression of *COMT2*, leading to significant increases in poplar plant height (10–30% increase) (Figure 3C), stem diameter (approximately 30% increase) (Figure 3D), and stem biomass (20–40% increase) (Figure 3F). Toluidine blue O staining of stem sections showed that fiber-cell-specific overexpression of *COMT2* promoted wood development (Figure 4A–F), with xylem proportion (Figure 4G), width (Figure 4H), and cell layer number (Figure 4I) increasing significantly in *P. tomentosa* overexpression lines compared to WT.

### 3.2. Specific Overexpression of COMT2 in Poplar Fiber Cells Promotes Secondary Wall Synthesis and Stem Strength

Additionally, the researchers found that COMT, as a key downstream enzyme in lignin biosynthesis, influences lignin biosynthesis and directly determines monolignol composition [27,28]. Our study demonstrated through phloroglucinol HCl staining (Figure 5A–F) and lignin content measurements (Figure 5G) that specific overexpression of *COMT2* in poplar fiber cells promoted stem lignin biosynthesis. Further analysis revealed significant upregulation of several key lignin biosynthetic pathway genes (Figure 5H–J), indicating that overexpression of *COMT2* promotes lignin deposition by activating downstream synthetic pathways. Additionally, in transgenic plants, the SCW thickness of wood fiber cells increased by 25–35% (Figure 6G), while that of vessel cells showed no significant changes (Figure 6H). Stem strength in poplar serves as a critical indicator for maintaining stem erectness during growth, facilitating water and nutrient transport under stress conditions, and determining the quality of wood deposition. Because lignin accumulation affects the rigidity of secondary walls and plant lodging resistance [29], we found that overexpression of *COMT2* in fiber cells could significantly enhance stem bending, puncture, and extrusion resistance (Figure 6I), providing a theoretical foundation for cultivating ecological forests capable of windbreak and sand-fixation. To validate the functional conservation of *COMT2* across poplar species, this study generated transgenic *Populus deltoides × Populus euramericana ‘Nanlin895’* lines harboring the DUFpro:*COMT2* construct (Figure S2 and Figure 7A). Consistent with phenotypes observed in *P. tomentosa* transgenic lines, specific upregulation of *COMT2* in fiber cells of *Populus deltoides×Populus euramericana ‘Nanlin895’* poplar promoted plant growth (Figure 7A–C), stem biomass accumulation (Figure 7D), stem lignin biosynthesis (Figure 7E–H), SCW deposition (Figure 7I–L) and stem mechanical strength (Figure 7M).

## 4. Materials and Methods

### 4.1. Plant Materials and Growth Conditions

*Populus tomentosa* and *Populus deltoides × Populus euramericana ‘Nanlin895’* were cultivated in a greenhouse under a 16 h light/8 h dark cycle, with the light period maintained at 25 °C and a photosynthetic photon flux density of 5000 lux, and the dark period at 23 °C. The relative humidity was consistently maintained at 60% throughout the cultivation period.

### 4.2. Phylogenetic Tree Construction and Sequence Analysis

Gene IDs corresponding were obtained from the National Genomics Data Center (NGDC; https://bigd.big.ac.cn/bioproject), accessed on 1 May 2022 and Phytozome (https://phytozome-next.jgi.doe.gov/blast-search), accessed on 1 May 2022. Phylogenetic trees were constructed using MEGA11, and conserved functional motifs in amino acid sequences were analyzed with DNAMAN (San Ramon, CA, USA) (Figure 1B).

### 4.3. Vector Construction and Plant Transformation

The full-length cDNA of the *COMT2* gene was amplified from *P. tomentosa* cDNA using gene-specific primers (Table S1) and cloned into a modified pCAMBIA2300 vector carrying a *DUF579-9* promoter. The *DUF*pro:*COMT2* construct was stably transformed to wild-type *P. tomentosa* and *Populus deltoides × Populus euramericana ‘Nanlin895’* plants through the method of *Agrobacterium*-mediated infiltration of leaf disks as described previously [30]. Positive transgenic lines were identified via PCR screening with gene-specific primers and subsequent kanamycin resistance selection.

### 4.4. RNA Extraction and Quantitative RT-PCR

Total RNA was extracted from transgenic plant stems using the Biospin Plant Total RNA Extraction Kit (Bioflux, Hangzhou, China). Reverse-transcribed into cDNA using a PrimeScriptTM RT reagent kit with gDNA Eraser (TaKaRa, Dalian, China), and real-time quantitative polymerase chain reaction (RT-qPCR) was conducted using SYBR Premix ExTaqTM (Takara) in a qTOWER3G IVD real-time PCR machine (Saale Valley, Jena, Germany). The poplar *Ubiquitin* gene (UBQ) was used as an internal standard. The primers used for RT-qPCR assays are listed in Table S1. The RT-qPCR expression data of target gene expression were calculated using the ΔΔCt method. Three biological and three technical replicates were performed for each gene to ensure reproducibility.

### 4.5. Cross-Sectioning and Histological Staining

Due to the differences in the number of internodes between the WT and *DUF*pro:*COMT2* transgenic plants, we marked the transgenic materials that had grown for one month; the third internode was marked every 10 days, with a total of four markings. Fresh stem cross-sections of the 2nd marked internodes of 2-month-old WT, *DUF*pro:*COMT2* transgenic poplars were cut into 70 μm cross-sections using a vibrating blade microtome (VT1000s, LEICA) (Wetzlar, HE, Germany) and stored in formalin–acetic acid–alcohol (FAA) fixative. The cross-sections were then stained with 0.05% (*w*/*v*) toluidine blue or phloroglucinol-HCl for 1–2 min and further observed and captured using a microscope (Zeiss) (Oberkochen, BW, Germany). The image data was measured and analyzed through IMAGEJ (Figure 4G–I, Figure 6G–I and Figure 7L) (https://imagej.nih.gov/ij/, accessed on 1 May 2022) (Bethesda, MD, USA) for quantifying morphological parameters of xylem cells.

### 4.6. Scanning Electron Micrograph (SEM) Observation

Fresh stem cross-sections of the 2nd marked internodes of 2-month-old WT, *DUF*pro:*COMT2* transgenic poplars were harvested as previously mentioned and fixed in FAA solution, then dehydrated using graded ethanol. Finally, the samples were transferred to a scanning electron microscopy chamber and imaged under BSE mode at 15 kV and then directly viewed under the microscope (Phenom^TM^ Pure FEI, Eindhoven, The Netherlands) following the manual’s recommendations. The images were captured digitally. The images were analyzed by IMAGEJ for quantifying the thickness of the secondary cell wall in fiber cells and vessel cells.

### 4.7. Lignin Content Determination

The stems from 2-month-old WT, *DUF*pro:*COMT2* transgenic poplars were harvested and dried at 60 °C. Dried samples were ground into powder that can pass through a 40-mesh sieve and kept dry until use. The content of total lignin was quantified using Klason, acid-soluble and acetyl bromide-soluble (AcBr) lignin methods (Boxbio, Beijing, China). This method utilizes a lignin content detection kit for measurement (Boxbio, Beijing, China). In the first step, 250 µL of reagent one, 3 mg of weighed sample, and 10 µL of perchloric acid are sealed and thoroughly mixed, followed by acetylation in an 80 °C water bath for 40 min, with gentle mixing every 10 min. After the reaction, the mixture is allowed to cool naturally to room temperature. In the second step, 250 µL of reagent two is added and thoroughly mixed. After standing at room temperature for a short period, the supernatant is collected and diluted with glacial acetic acid. The OD at 280 nm is measured, with the dilution ratio adjusted to ensure the OD value falls within the range of 0.1 to 0.8. The lignin content is determined based on the conversion formula. Three biological replicates were performed in each experiment.

### 4.8. Stem Strength Determination

Mechanical resistance to bending, puncture, and extrusion was tested on basal internodes of 2-month-old WT and *DUF*pro:*COMT2* transgenic poplar stems. Sample lengths were standardized at 2 cm (bending), 1 cm (puncture), and 1 cm (extrusion) with consistent measurement conditions. Three biological replicates were performed for the test, and average force values were calculated.

### 4.9. Accession Numbers

The sequences used in this study are available in the National Genomics Data Center and Phytozome under the following accession numbers: Potri.012G006400.1; LOC_Os08g06100.1; Podel.05G175000.1; Potri.015G003100.1; LOC_Os04g09654.1; Podel.15G003000.1; AT5G54160.1; P.x_tomentosa74519.t1; AT1G77520.1; P.x_tomentosa72428.t1.

## Data Availability

The original contributions presented in this study are included in the article/Supplementary Material. Further inquiries can be directed to the corresponding author.

## Supplementary Materials

The following supporting information can be downloaded at https://www.mdpi.com/article/10.3390/plants14121739/s1, Figure S1. Identification of *COMT2* overexpression lines in *P.tomentosa.* Figure S2. Identification of *COMT2* overexpression lines. Figure S3. *COMT2*-dependent regulation on xylem cell development during wood formation in *P.deltoides* × *P. euramericana cv ‘Nanlin895’.* Figure S4. *COMT2* regulates the expression of lignin biosynthetic genes in *P.deltoides × P. euramericana cv ‘Nanlin895’*. Table S1. Sequences of oligonucleotide primers and probes used in this study.
