# Supplementary material for: The Fiber Cell-Specific Overexpression of COMT2 Modulates Secondary Cell Wall Biosynthesis in Poplar
Source: Plants (Basel). 2025 Jun 6;14(12):1739. doi: 10.3390/plants14121739 (PMC12197300; doi:10.3390/plants14121739)
Supplement: Supplementary file 1 [file plants-14-01739-s001.zip › Supplemental data-2025.6.5 .pdf]

SUPPLEMENTAL FIGURES

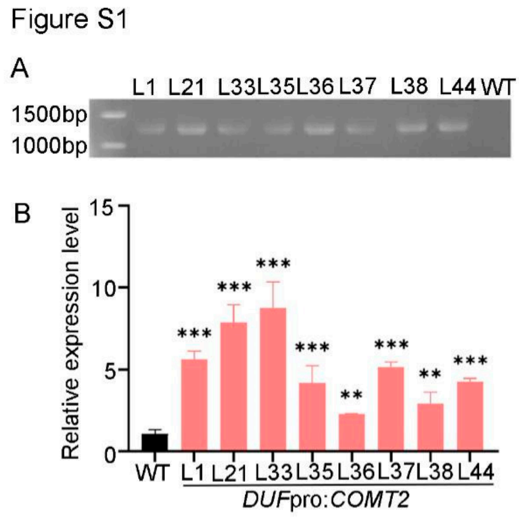

Figure S1. Identification of *COMT2* overexpression lines in *P.tomentosa*

A. Identification of *DUF*pro:*COMT2* transgenic lines in *P.tomentosa*. B. Expression level of COMT2 in WT and independent transgenic lines in *P.tomentosa*. The poplar UBQ gene was used as an internal control.Student's *t*-test:** $p < 0.01$; *** $p < 0.001$; $n = 3$.

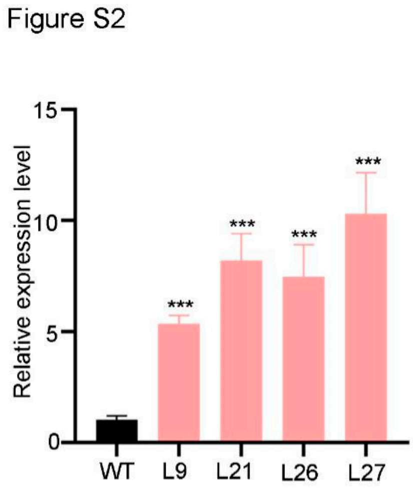

Figure S2. Identification of *COMT2* overexpression lines

Expression level of COMT2 in WT and independent transgenic lines in *P deltoides* × *P. euramericana* cv 'Nanlin895'. The poplar UBQ gene was used as an internal control. Student's *t*-test:*** $p < 0.001$; $n = 3$.

Figure S3

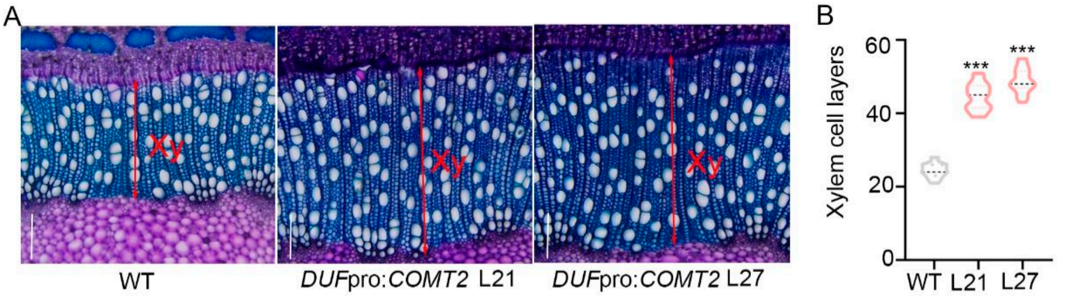

Figure S3. ***COMT2*****-dependent regulation on xylem cell development during wood formation in *P.deltoides* × *P. euramericana* cv 'Nanlin895'**

(A) Cross sections of the marked stem internode were stained with toluidine blue from 2-month-old WT and *DUF*pro:*COMT2 P.deltoides* × *P. euramericana* cv 'Nanlin895' transgenic plants. Bar=200 µm. (B) Measurement and quantification of secondary xylem cell layers.Student's *t*-test:*** $p < 0.001$; $n = 3$. Xy means xylem.

Figure S4

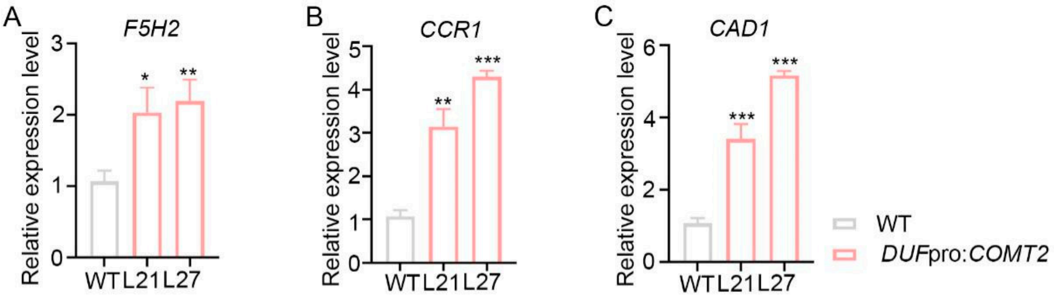

Figure S4. ***COMT2*** **regulates the expression of lignin biosynthetic genes in *P.deltoides* × *P. euramericana* cv 'Nanlin895'**

(A-C) Expression analysis of lignin biosynthetic genes in WT and *DUF*pro:*COMT2 P.deltoides* × *P. euramericana* cv 'Nanlin895' transgenic plants, including *F5H2* (A), *CCR1* (B) and *CAD1* (C). Student's t-test: *, $P<0.05$; **, $P<0.01$; ***, $P<0.001$; n=3.

Table S1. Sequences of oligonucleotide primers and probes used in this study

Name	Sequence
**COMT2 (DUF)-F**	5'TATATAAGGAAGTTGGTACCATGGGTTCGACAGGTGAAAC3'
**COMT2 (DUF)-R**	5'TGTTTGACTAGTCGCTGCAGTTAGTTCTTGCGGAATTCAATG3'
**DUF579-9-F**	5' GTCTTCCCGCTGCACTACTC3'
**DUF579-9-R**	5' AAGGAGTCGTTTGGCCAATC3'
**UBQ-F**	5'GTTGATTTTTGCTGGGAAGC3'
**UBQ-R**	5'GATCTTGGCCTTCACGTTGT3'
**qCOMT2 -F**	5'GCCCTGGTGCTTTCTTGT3'
**qCOMT2 -R**	5'CCGTCCTCGTTCTTGGTC3'
**qF5H2-F**	5'GAGTCCAGCAAGAGCTCGCAG3'
**qF5H2-R**	5'GCATAAGCATTGATCATCAC3'

**qCAD1-F**	5’CAAGCTGATCTTGATGGGTG3’
**qCAD1-R**	5’CGAATCTATATCTCACATC3’
**qCCR-F**	5’CTGTTCAAGCTTATGTGCATG3’
**qCCR-R**	5’GTGGAGAACGCTCTCAGAGC3’
