# Supplementary figures and images for: The Fiber Cell-Specific Overexpression of COMT2 Modulates Secondary Cell Wall Biosynthesis in Poplar
Source: Plants (Basel). 2025 Jun 6;14(12):1739. doi: 10.3390/plants14121739 (PMC12197300; doi:10.3390/plants14121739)

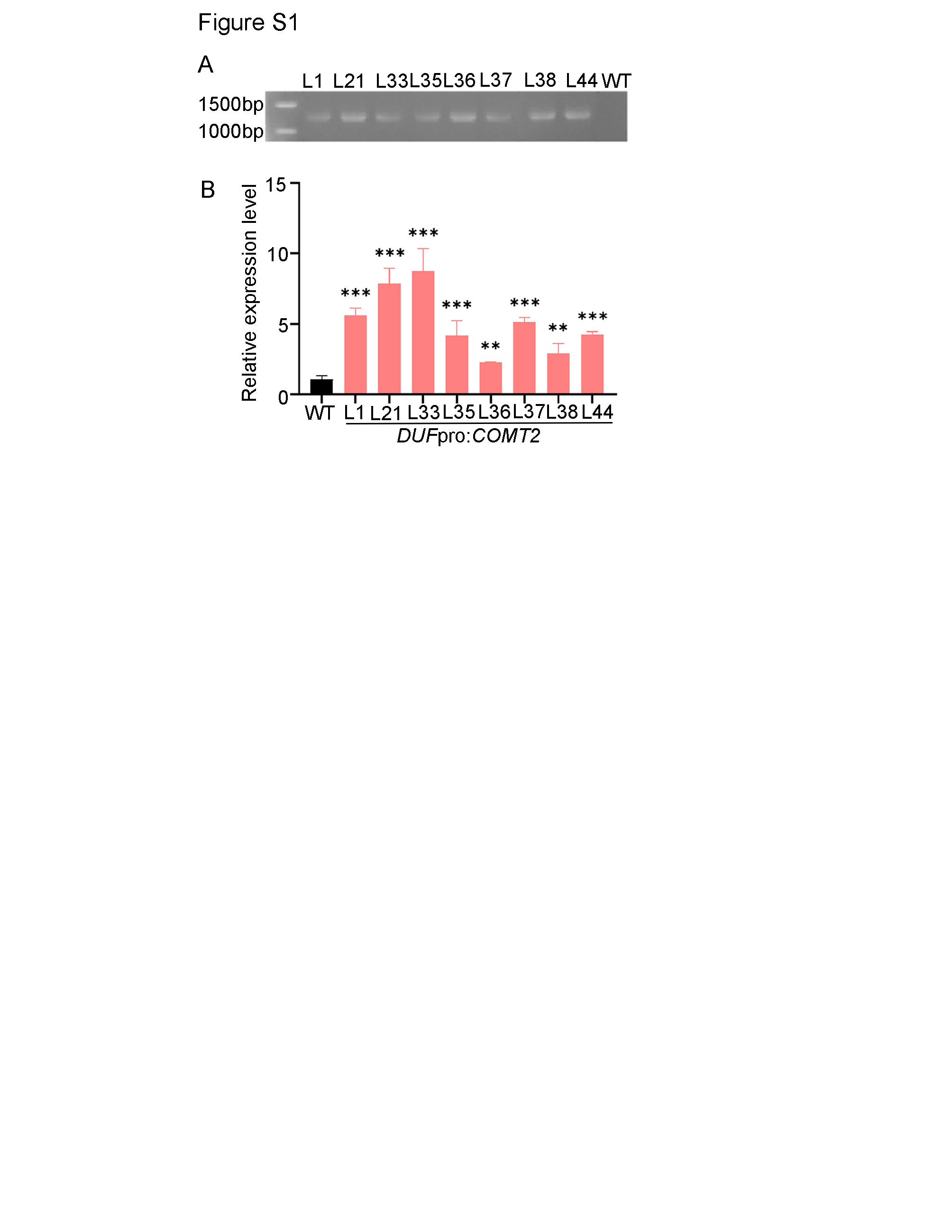

Supplement: Supplementary file 1 [file plants-14-01739-s001.zip › Figure S1.jpg]

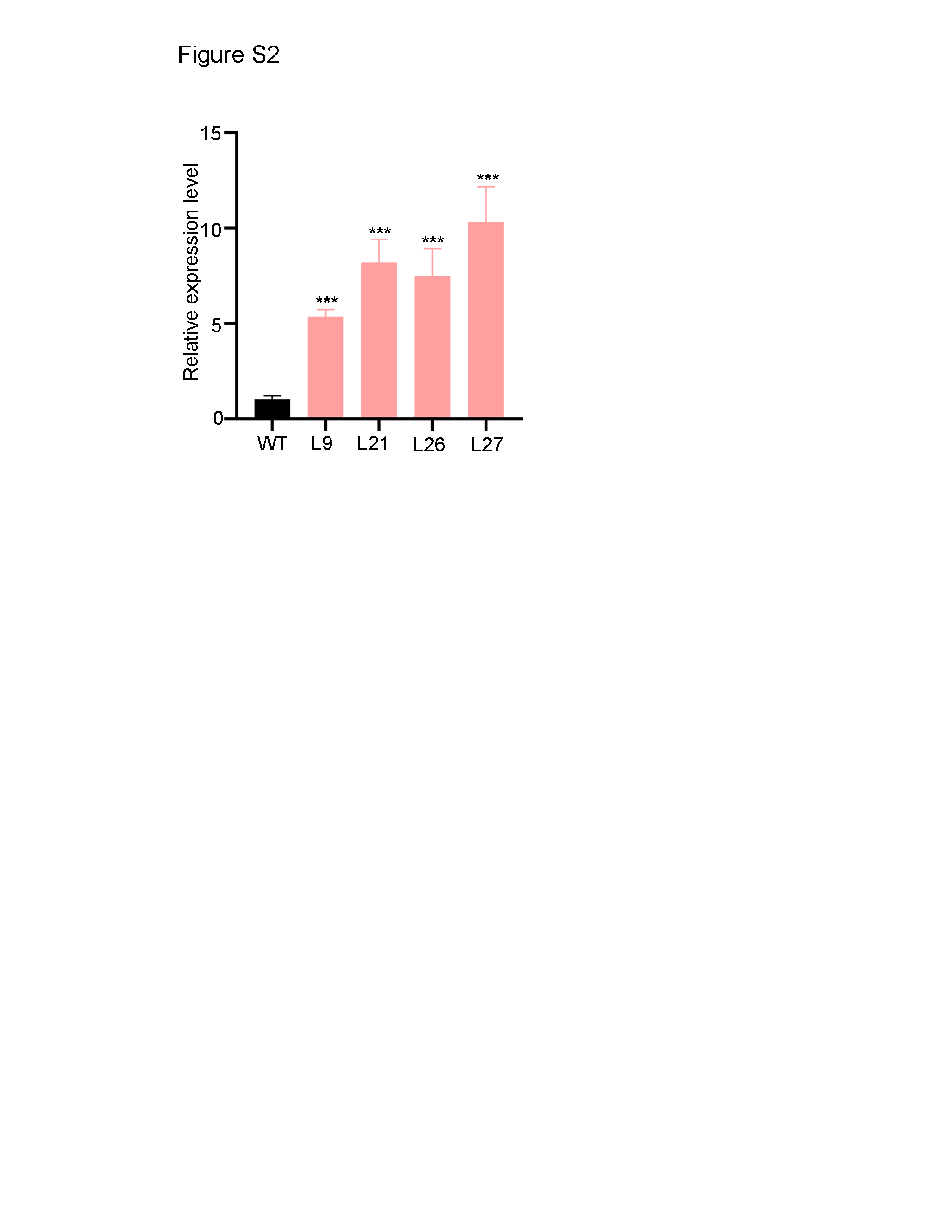

Supplement: Supplementary file 1 [file plants-14-01739-s001.zip › Figure S2.jpg]

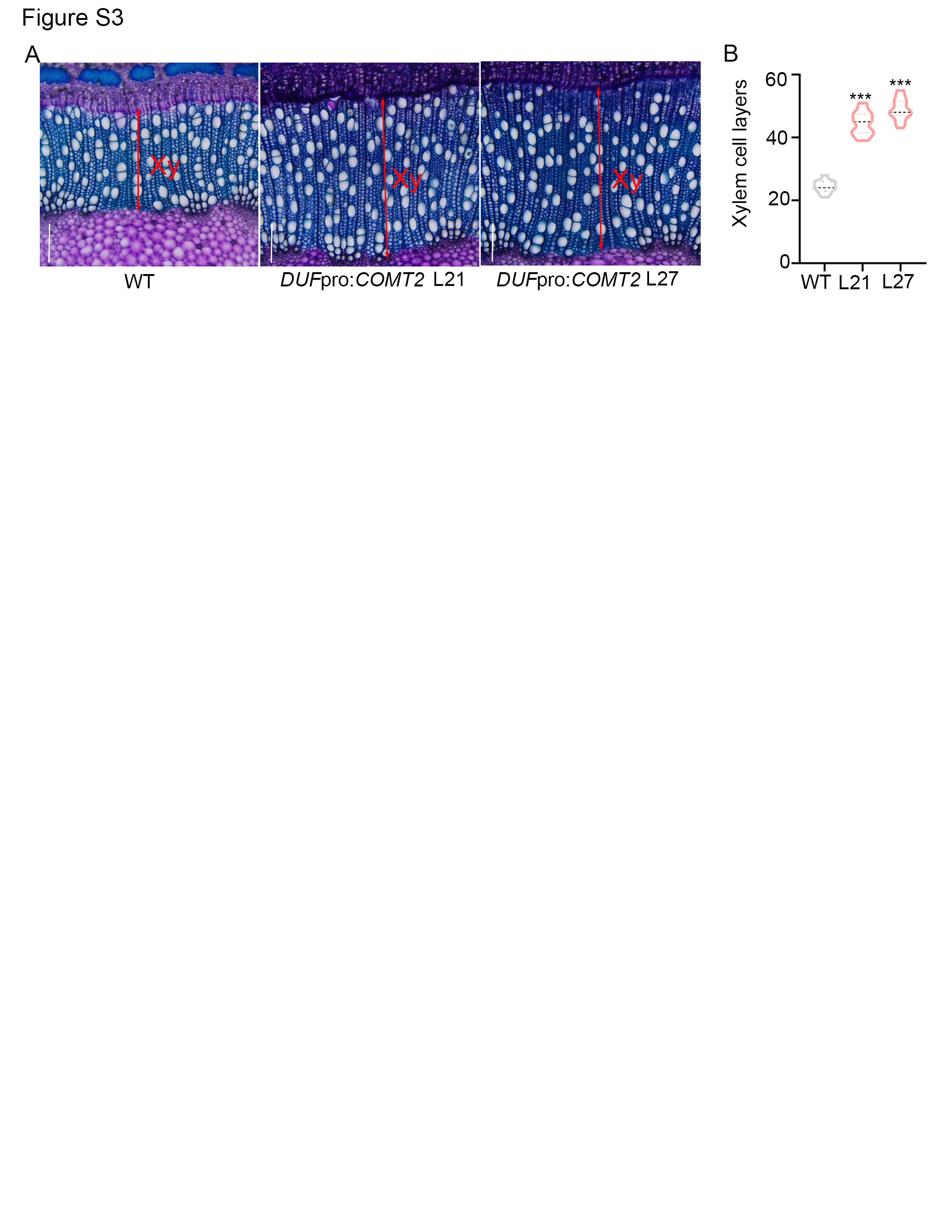

Supplement: Supplementary file 1 [file plants-14-01739-s001.zip › Figure S3.jpg]

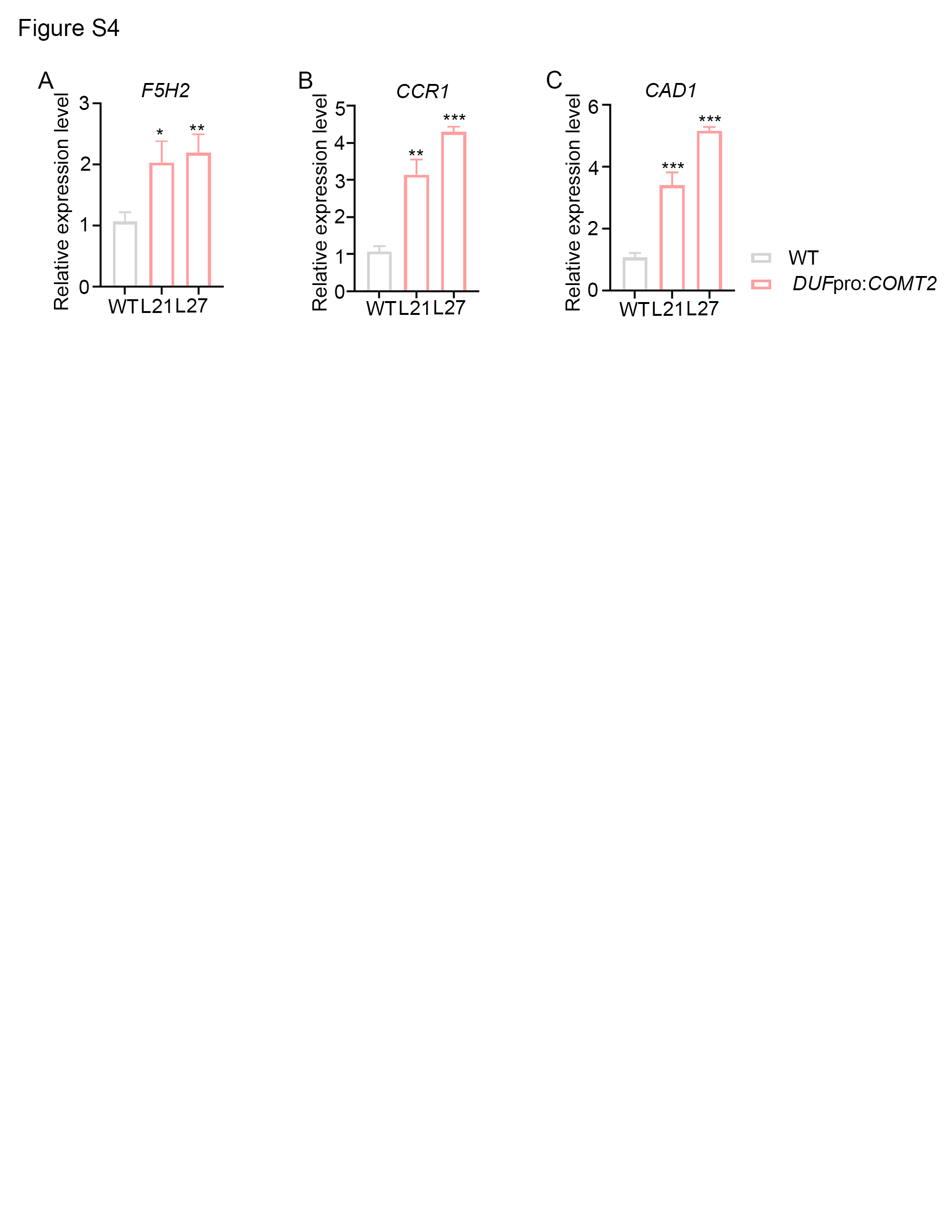

Supplement: Supplementary file 1 [file plants-14-01739-s001.zip › Figure S4.jpg]
